# Breaking down relationship barriers to increase PrEP uptake and adherence among adolescent girls and young women in Kenya: safety and preliminary effectiveness results from a pilot cluster‐randomized trial

**DOI:** 10.1002/jia2.26198

**Published:** 2023-12-20

**Authors:** Sarah T. Roberts, Miriam Hartmann, Alexandra M. Minnis, Sophie Odek Otticha, Erica N. Browne, Elizabeth T. Montgomery, Kawango Agot

**Affiliations:** ^1^ Women's Global Health Imperative RTI International Berkeley California USA; ^2^ Impact Research and Development Organization Kisumu Kenya

**Keywords:** adolescent girls and young women, HIV prevention, pre‐exposure prophylaxis, adherence, Africa (region), violence

## Abstract

**Introduction:**

Oral pre‐exposure prophylaxis (PrEP) has the potential to reduce HIV acquisition among adolescent girls and young women (AGYW) in sub‐Saharan Africa, a priority population for epidemic control. However, intimate partner violence (IPV) and low relationship power can create significant challenges to PrEP use. The Tu'Washindi intervention aimed to increase PrEP use by addressing relationship‐ and violence‐related barriers among AGYW enrolled in the DREAMS Initiative in Siaya County, Kenya.

**Methods:**

Our multi‐level, community‐based intervention was piloted in a cluster‐randomized controlled trial conducted at six DREAMS sites from April to December 2019 (NCT03938818). Three intervention components were delivered over 6 months: an eight‐session empowerment‐based support club, community sensitization targeted towards male partners and a couples’ PrEP education event. Participants were ages 17–24, HIV negative and either eligible for, or already taking, PrEP. Over 6 months of follow‐up, we assessed IPV (months 3 and 6) and PrEP uptake and continuation (month 6) through interviewer‐administered questionnaires; PrEP adherence was assessed with Wisepill electronic monitoring devices. These outcomes were compared using adjusted Poisson and negative binomial regression models.

**Results:**

We enrolled 103 AGYW with median age of 22 years (IQR 20–23); one‐third were currently taking PrEP and 45% reported IPV in the past 3 months. Retention was 97% at month 6. Compared to the control arm, intervention arm participants were more likely to initiate PrEP, if not already using it at enrolment (52% vs. 24%, aRR 2.28, 95% CI 1.19–4.38, *p* = 0.01), and those taking PrEP had more days with device openings (25% of days vs. 13%, aRR 1.94, 95% CI 1.16–3.25, *p* = 0.01). Twenty percent of participants reported IPV during follow‐up. There were trends towards fewer IPV events (aIRR 0.66, 95% CI 0.27–1.62, *p* = 0.37) and fewer events resulting in injury (aIRR 0.21, 95% CI 0.04–1.02, *p* = 0.05) in the intervention versus control arm.

**Conclusions:**

Tu'Washindi shows promise in promoting PrEP uptake and adherence among AGYW without concomitant increases in IPV; however, adherence was still suboptimal. Further research is needed to determine whether these gains translate to increases in the proportion of AGYW with protective levels of PrEP adherence and to evaluate the potential for the intervention to reduce IPV risk.

## 1 INTRODUCTION

Adolescent girls and young women (AGYW; ages 15–24) in sub‐Saharan Africa are disproportionately affected by HIV. More than one in four new HIV acquisitions in this region are among AGYW [1], who are over twice as likely to acquire HIV as their male counterparts [2]. Oral pre‐exposure prophylaxis (PrEP) is a proven biomedical HIV prevention intervention, but its protective benefit has been limited among AGYW across sub‐Saharan Africa by challenges to uptake, adherence and persistence [3, 4, 5, 6, 7, 8, 9, 10, 11, 12, 13, 14, 15, 16, 17, 18]. In western Kenya, the proportion of AGYW persisting with PrEP for 3 months ranges from 5% to 37% [5, 8, 11, 12, 17], and in a cross‐sectional study of PrEP users, only 6% had biomarker levels, suggesting high adherence [7]. The public health impact of PrEP depends on concurrent interventions that address critical barriers to uptake and adherence.

AGYW who live in a context of heightened gender inequality and intimate partner violence (IPV) risk represent a large sub‐population who are uniquely vulnerable to HIV acquisition and face exacerbated barriers to PrEP use [19, 20, 21, 22, 23, 24]. Over one in three women worldwide experience IPV, and experience or fear of IPV is associated with having limited relationship power and with 28%–55% higher HIV incidence [25, 26, 27, 28]. Violence and other partner‐related social harms are associated with 1.5‐ to 2.5‐fold higher risk of poor adherence to oral PrEP and the dapivirine ring for HIV prevention [19, 22, 29–32]. As with HIV treatment and contraception [26, 33–39], IPV and relationship inequality introduce barriers to the uptake of and adherence to PrEP across multiple socio‐ecological levels. Barriers include restricted information and access, low self‐efficacy and fear of relationship dissolution at the individual level [40, 41, 42]; disclosure challenges and low decision‐making power at the partner level [43, 44, 45]; and lack of PrEP awareness, inequitable gender norms and PrEP stigma at the community level [46, 47, 48, 49].

In light of these challenges, we partnered with AGYW in Siaya County, Kenya to develop and pilot test *Tu'Washindi na PrEP* (We're Winners with PrEP), a multilevel, community‐based intervention that aims to increase PrEP use by addressing relationship‐ and violence‐related challenges. In this paper, we present pilot study results on intervention safety (i.e. IPV and social harms) and effectiveness on PrEP uptake, continuation and adherence.

## 2 METHODS

### 2.1 Study setting

All study activities were nested in the DREAMS programme in Siaya County, Kenya, where annual HIV incidence among AGYW is 5 per 1000 person ‐years [50]. IPV is also widespread in this region, with 19% of AGYW reporting experience of sexual IPV in the past year, 25% physical IPV and 34% emotional IPV [51]. DREAMS is a public‐private partnership aiming to reduce HIV incidence among AGYW through key activities, including school and community‐based HIV and IPV prevention; referrals for post‐violence care; re‐enrolment of girls in schools; and provision of HIV counselling and testing, condoms and contraception, PrEP and social asset building through “Safe Spaces” [52]. Safe Spaces are community locations where groups of AGYW convene weekly for life skills education led by a female mentor. PrEP and other sexual health services are provided by a mobile team of clinicians and counsellors who visit the Safe Spaces on a rotating schedule.

### 2.2 The *Tu'Washindi* intervention

DREAMS initiated over 4000 AGYW on PrEP in Siaya County from 2017 to 2020, representing approximately 15% of PrEP‐eligible participants, with about 35% continuation at month 1. To improve PrEP uptake and adherence, we used participatory methods engaging local AGYW and DREAMS providers to co‐design an intervention that could be easily integrated into ongoing DREAMS activities and deliver supplemental, targeted content. The intervention included three components delivered over 6 months to different targets:

1. AGYW: An eight‐session empowerment‐based Support Club. Sessions occurred biweekly for the first 2 months and monthly thereafter, and were facilitated by a Safe Space mentor or by a participant with mentor support. DREAMS clinicians and counsellors attended 2–3 sessions each to lead specific activities.
2. Male partners of AGYW: Community sensitization events, led by male “Change Agents” from the SASA! programme (delivered though DREAMS) [53], with support from DREAMS clinicians. Events were scheduled weekly over the first 3 months in places where AGYW's male partners are known to gather (e.g. boda boda [motorbike taxi] drivers’ meetings, chief's meetings [barazas]).
3. AGYW and their male partners: Couples’ PrEP education events called “Buddy Days” were held at each intervention Safe Space in month 3 of the intervention period, co‐facilitated by the Change Agent and clinician. To destigmatize participation, Buddy Days were open to all community members and offered routine health services. All AGYW and their partners who attended the Buddy Day together as a couple received a small basket of foodstuffs worth approximately $4 USD.

Additional information about the development, theoretical basis and content of Tu'Washindi has been previously published [54]. The intervention was manualized and the study coordinator trained each cadre of providers (i.e. mentors, Change Agents, clinicians and counsellors) on relevant content and responsibilities before implementation. Fidelity and quality of each intervention activity were assessed by a research assistant using a structured form.

### 2.3 Pilot study design, participants and procedures

The intervention was pilot‐tested in a cluster‐randomized controlled trial from April to December 2019. The primary outcomes were feasibility and acceptability (reported elsewhere) and safety. Secondary outcomes of PrEP uptake, adherence and continuation were assessed to gather preliminary evidence of efficacy, but it was not possible to power the study for these outcomes in this pilot stage. Six geographically separate DREAMS Safe Spaces were selected, matched into pairs based on size, geographic setting (peri‐urban, rural or fishing) and the proportion of attendees potentially eligible for PrEP, then randomized 1:1 within pairs to the intervention plus usual DREAMS services or usual DREAMS services alone. Participants were recruited during routine Safe Space meetings. Eligibility criteria included: aged 15–24; enrolled in DREAMS and attended at least one activity at the Safe Space in the past 3 months; HIV negative by self‐report; met behavioural eligibility criteria for PrEP, as defined by the 2016 Kenya national guidelines [55]; potentially interested in PrEP or already using PrEP; and willing and able to attend support groups over the 6‐month study period. Behavioural and socio‐demographic data were collected from enrolled participants via face‐to‐face interviews with trained research assistants at three time points: baseline, month 3 (intervention mid‐point) and month 6 (post‐intervention). All interviews were conducted in the participant's preferred language (English, Dholuo or Kiswahili) in a private setting.

### 2.4 Measures

*Participant characteristics* included socio‐demographic factors, partner and relationship characteristics, sexual behaviour and HIV risk perception.

*PrEP uptake* was defined as initiating PrEP during the study period, among those not on PrEP at enrolment. It was assessed by self‐report and confirmed with records from the DREAMS Health Management Information System (HMIS).

*PrEP continuation* was defined as continuing to take PrEP at study exit. Participants who reported any PrEP use were asked the date of initiation, if they were still taking PrEP, and if applicable, the month, year and reason for discontinuation. We assumed that discontinuation occurred on the first day of the month reported by the participant.

*PrEP adherence* was assessed using Wisepill, an electronic adherence monitoring device (Wisepill Technologies, South Africa). All device openings were automatically captured and downloaded from the device to a central server. Adherence was defined as the number of days with a Wisepill opening during the self‐reported period of PrEP use.

*Safety assessments*: Physical, sexual and emotional IPV were assessed at every visit using 13 items from the World Health Organization Violence Against Women survey [56], plus one additional question on non‐consensual sex due to pressure without threats of physical harm [57]. Our primary safety outcome was the number of “reportable” IPV episodes during the study period, defined as any IPV except one‐time experiences of moderate emotional violence [58]. A secondary outcome was the number of severe events, defined as reportable episodes that resulted in physical injury.

*Study‐related social harms* were assessed prospectively by asking if the participant had experienced any negative change, event or experience in her life that was related to her study participation. Social harms could also be reported by participants between study visits. Negative experiences attributable to PrEP use were also considered social harms since the study encouraged participants to initiate or continue PrEP.

### 2.5 Analysis

We calculated descriptive statistics for baseline socio‐demographic characteristics, by study arm, to assess whether balance was achieved by randomization. All outcome assessments use an intent‐to‐treat approach, comparing participants by study arm. All regression models accounted for clustering by DREAMS Safe Space with adjustment for matched site pairs as a fixed effect.

PrEP uptake and continuation were compared across study arms using Poisson regression with robust standard errors. The PrEP continuation model controlled for time since PrEP initiation, to account for evidence from other studies that adherence declines significantly over time [5, 59, 60]. For PrEP adherence, we report on the proportion of participants with high adherence, defined as Wisepill openings on >85% of days that PrEP was used during the study. This cut‐off is based on pharmacodynamic modelling, suggesting that six doses of oral PrEP per week may be required to protect female genital tissue from HIV infection [61]. High adherence was compared descriptively by arm due to small cell sizes. We also compared the number of days adherent by arm using negative binomial regression, controlling for the duration of PrEP use.

IPV outcomes were compared by arm with Poisson regression with robust standard errors. Reports of social harm were summarized descriptively due to the small number of events.

#### 2.5.1 Subgroup analysis

We conducted a post‐hoc exploratory subgroup analysis to establish whether intervention effects were comparable among women with previous experience of IPV. All regression analyses were repeated in the subgroup of women reporting any history of IPV from their current or most recent partner at baseline.

### 2.6 Ethics

This study was approved by the Maseno University Ethics Review Committee in Kenya (MUERC; protocol #MSU/DRPI/MUERC/00418/17). RTI International agreed to rely on MUERC's ethical oversight through a signed IRB Authorization Agreement. All participants were adults or emancipated minors and provided written informed consent in their preferred language. The study is registered with Clinical trials.gov (NCT03938818).

## 3 RESULTS

**Figure 1:**
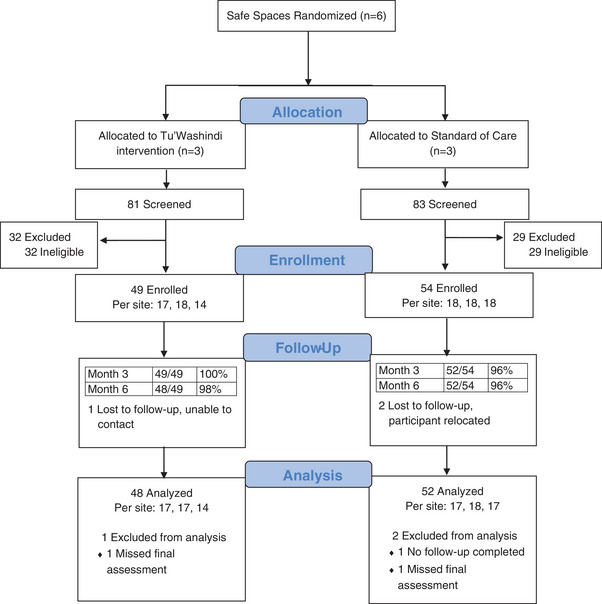
Study CONSORT diagram.

Screening, enrolment and retention are described in the Figure 1 CONSORT diagram. We screened 164 participants and enrolled 103 (63%): 49 participants in the intervention arm, and 54 in the control arm. The main reasons for study ineligibility included not eligible for PrEP (70%), not interested in PrEP (48%), not willing to comply with the study requirements (25%) and living with HIV (18%; participants could be ineligible for multiple reasons). Retention was 98% (101/103) at the month‐3 safety visit and 97% (100/103) at the month‐6 follow‐up/exit visit, and was similar across arms.

### 3.1 Participant characteristics

At baseline, the median age of participants was 22 years (range: 17–24). About half had any secondary school education (53%) and were married (56%) or cohabiting (54%; Table 1). Sixty percent (60%) reported any history of physical, sexual or emotional IPV from their current or most recent partner, including 45% in the past 3 months; 40% reported any history of financial abuse. Forty‐six percent had ever used PrEP and 32% were currently using it. Nearly half reported that their primary partners were aware of their PrEP use or their interest in using PrEP (47%); among these participants, 58% said their partner was supportive.

**Table 1:** Baseline characteristics, by study arm

	Intervention		Control		Total	
	*N*	%	*N*	%	*N*	%
Total	49	100	54	100	103	100
**Demographics**						
Age (median, IQR)	22 (20−23)		22.5 (21−24)		22 (20−23)	
Any secondary education	29	59	26	48	55	53
Currently in school	8	16	7	13	15	15
Married	26	53	32	59	58	56
Number of children (median, IQR)	1 (1−2)		2 (1−2)		2 (1−2)	
Employed	13	27	17	32	30	29
Any food insecurity, past 4 weeks	25	51	30	56	55	53
Attends DREAMS Safe Space at least once/week	44	90	48	89	92	89
**Male partner and relationship characteristics**						
Number of partners, past 6 months (median, IQR)	1 (1–1)		1 (1–2)		1 (1–2)	
Has a primary partner	46	94	52	96	98	95
Age difference with primary partner (median, IQR)^a^	4 (2–6)		4 (2–6)		4 (2–6)	
Lives with male partner	23	47	33	61	56	54
Male partner provides financial support	36	73	46	87	82	80
**Abuse from current or most recent primary partner**						
Any financial abuse, lifetime	19	39	22	41	41	40
Any IPV (physical, sexual, emotional), lifetime	30	61	32	59	62	60
Past 3 months	23	47	23	43	46	45
Any emotional violence, lifetime	26	53	26	48	52	50
Past 3 months	19	39	20	37	39	38
Any physical violence, lifetime	20	41	23	43	43	42
Past 3 months	13	27	15	28	28	27
Any sexual violence, lifetime	16	33	19	35	35	34
Past 3 months	9	18	14	26	23	22
**PrEP use and partner disclosure/support**						
PrEP use, ever	22	45	25	46	47	46
Currently on PrEP	15	31	18	33	33	32
Partner aware of study participation	19	40	27	50	46	45
Partner aware of PrEP use/interest in PrEP	16	33	32	59	48	47
Partner supportive of PrEP use/interest in PrEP (*n* = 48)	10	63	18	56	28	58

Abbreviations: IPV, intimate partner violence; IQR, interquartile range; PrEP, pre‐exposure prophylaxis.

^a^ Primary partner's age in years—participant's age in years. Data were missing for three participants (two unknown partner age and one implausible value).

### 3.2 Intervention delivery

All support club sessions (8 per site) and buddy days (1 per site) were conducted as planned. Although 10 community sensitization activities were planned per site, only 4–6 occurred, due to challenges the Change Agents experienced in planning these events. Among 49 intervention arm participants, 100% attended ≥1 support club, and the mean number of sessions attended was 5.2 (standard deviation [SD] 2.0). Forty‐four participants (90%) attended the Buddy Days, including 35 (80%) with their partners. Fifteen AGYW (31%) reported that their partner attended a community sensitization session. Staff observations indicated high fidelity and quality of intervention delivery (Table S1).

### 3.3 PrEP uptake

Among 67 participants who were not on PrEP at enrolment, 25 (37%) initiated PrEP during the study. Uptake was significantly higher in the intervention arm than in the control arm (51.5% vs. 23.5%; adjusted risk ratio [aRR] 2.28; 95% CI 1.19–4.38; *p* = 0.01; Table 2).

**Table 2:** Primary PrEP outcomes: intent‐to‐treat analysis

PrEP uptake^a^		*N*	*n* (%)	aRR^b^	95% CI	*p*
Initiated PrEP during the study	Intervention	33	17 (51.5%)	2.28	1.19–4.38	0.01
	Control	34	8 (23.5%)	ref		
**PrEP continuation** ^c^						
Taking PrEP at study exit	Intervention	32	26 (81.3%)	1.05	0.83–1.33	0.68
	Control	26	21 (80.7%)	ref		
**PrEP adherence, continuous** ^c^, ^d^		**Mean (SD) days of use**	**Mean (SD) days opened**	**aIRR** ^b^	**95% CI**	***p***
Number of days with Wisepill opening	Intervention	156.4 (48.0)	42.4 (53.0)	1.94	1.16–3.25	0.01
	Control	167.3 (57.3)	22.8 (27.4)	ref		

^a^ Among those not already on PrEP at enrolment (*n* = 67).

^b^ All effect estimates are adjusted for matched pairs of study sites. The estimate for PrEP continuation is additionally adjusted for time since PrEP initiation. The estimate for the number of days of Wisepill opening is additionally adjusted for the number of days the participant was on PrEP and had a Wisepill device in her possession.

^c^ Among those with any PrEP use during the study, that is on PrEP at enrolment or initiated during study (*n* = 58).

^d^ Wisepill device data are missing or uninterpretable for five participants; *n* = 53 (intervention arm *n* = 29; control arm *n* = 24).

#### 3.3.1 PrEP continuation

Fifty‐eight participants (56%) reported any PrEP use during the study, of whom 47 (81%) were continuing on PrEP at study exit. The main reasons for discontinuation were no longer at risk (*n* = 5) and unable to contact the clinician (*n* = 4). Mean duration of PrEP use from initiation (before or after study enrolment) to discontinuation or study exit was 379 days (SD: 365) in the control arm and 273 days (SD: 273) in the intervention arm. The probability of continuation, adjusting for time since PrEP initiation, was similar between arms (aRR 1.05, 95% CI 0.83–1.33, *p* = 0.68; Table 2).

### 3.4 PrEP adherence

Among the 53 participants with available adherence data, the mean number of days on PrEP was 156.4 (SD 48.0) in the intervention arm and 167.3 (SD 57.3) in the control arm. Only three participants (5.7%) had high PrEP adherence over the duration of PrEP use, all of whom were in the intervention arm, so we were not able to estimate intervention effects. The mean number of days with a Wisepill opening over the duration of PrEP use was 42.4 (SD 53.0) in the intervention arm and 22.8 (SD 27.4) in the control arm, corresponding to 24.9% and 13.0% of the number of days on PrEP, respectively (adjusted incident rate ratio [aIRR] 1.94; 95% CI 1.16–3.25; *p* = 0.01; Table 2). The intervention effect was similar among participants who started PrEP during the study and those who were already on PrEP at enrolment (Table S2).

### 3.5 Intimate partner violence and other safety outcomes

Twenty‐one participants reported an IPV event during follow‐up, including 10 participants reporting 11 IPV events in the intervention arm and 11 participants reporting 18 IPV events in the control arm, a difference that was not statistically significant (aIRR 0.66, 95% CI: 0.27–1.62, *p* = 0.37; Table 3). There were fewer events causing physical injury in the intervention arm (2 events) than in the control arm (11 events; aIRR = 0.21, 95% CI 0.04–1.02, *p* = 0.05; Table 3). One social harm was reported during the study—an intervention arm participant reported emotional and physical IPV resulting from her partner's opposition to PrEP use. She resolved the incident without help from the study staff and gained her partners’ support.

**Table 3:** Primary IPV outcomes: intent‐to‐treat analysis

Any IPV		*N*	Number of events	aIRR^a^	95% CI	*p*
Number of reportable events^b^	Intervention	48	11	0.66	0.27–1.62	0.37
	Control	52	18	Ref		
**Severe IPV**						
Number of events resulting in physical injury	Intervention	48	2	0.21	0.04–1.02	0.05
	Control	52	11	Ref		

^a^ All effect estimates are adjusted for matched pairs of study sites.

^b^ We defined a case of “reportable” IPV as any violence except for one‐time reports experiences of “moderate” emotional violence, that is being insulted or humiliated by a partner.

### 3.6 Subgroup analysis

All results were similar in magnitude and direction among participants who reported any history of IPV at baseline. However, the estimated intervention effects were stronger in this subgroup than in the entire study sample for both PrEP uptake (aRR 4.68, 95% CI 1.64–13.33 *p* = 0.004) and IPV incidence (aIRR 0.39, 95% CI 0.15–0.99, *p* = 0.05; (Table 4).

**Table 4:** Exploratory analysis: results among the subgroup of women with any IPV history at baseline (*n* = 59)

PrEP uptake^a^		*N*	*n* (%)	aRR^b^	95% CI	*p*
Initiated PrEP during the study	Intervention	16	9 (56.3)	4.68	1.64–13.33	0.004
	Control	20	3 (15.0)	Ref		
**PrEP continuation** ^c^		***N***	***n* (%)**	**aRR** ^b^	**95% CI**	***p***
Taking PrEP at study exit	Intervention	22	19 (86.4)	0.98	0.76–1.27	0.90
	Control	13	12 (92.3)	ref		
**PrEP adherence, continuous** ^c^		**Mean (SD)** **days of use**	**Mean (SD)** **days opened**	**aIRR** ^b^	**95% CI**	***p***
Number of days with Wisepill opening	Intervention	144.0 (60.3)	40.9 (56.0)	1.49	0.76–2.93	0.25
	Control	176.9 (41.1)	28.9 (26.5)	Ref		
**Any IPV**		***N***	**Number of events**	**aIRR** ^b^	**95% CI**	***p***
Number of reportable events^d^	Intervention	29	8	0.39	0.15–0.99	0.05
	Control	30	18	Ref		
**Severe IPV**		***N***	**Number of events**	**aRR** ^b^	**95% CI**	***p***
Number of events resulting in physical injury	Intervention	29	0	*Not estimable*		
	Control	30	11			

^a^ Among those not already on PrEP at enrolment (*n* = 36).

^b^ All effect estimates are adjusted for matched pairs of study sites. The estimate for PrEP continuation is additionally adjusted for time since PrEP initiation. The estimates for the number of days of Wisepill opening is additionally adjusted for the number of days the participant was on PrEP and had a Wisepill device in her possession.

^c^ Among those with any PrEP use during the study, that is on PrEP at enrolment or initiated during study (*n* = 35; intervention arm *n* = 22; control arm *n* = 13).

^d^We defined a case of “reportable” IPV as any violence except for one‐time reports experiences of “moderate” emotional violence, that is being insulted or humiliated by a partner.

## 4 DISCUSSION

This study sought to assess whether a multilevel, community‐based intervention comprised of support clubs, male partner sensitization and a couples’ education session showed promise to increase PrEP uptake and adherence among AGYW in Kenya without concomitant risks to their safety. Results indicate that intervention participants had approximately two‐fold higher PrEP uptake and adherence than control arm participants, and no evidence of increased IPV or social harms. We also observed significantly lower rates of IPV in the intervention arm among participants with a prior history of IPV, suggesting that the intervention might also be an effective IPV reduction strategy. However, PrEP adherence remained low among intervention arm participants, suggesting that they faced additional barriers not addressed by Tu'Washindi, and that the intervention may need to be delivered alongside other PrEP promotion programmes.

To our knowledge, this is the first intervention to show preliminary evidence of increasing PrEP uptake and adherence in a controlled trial. Several scoping/systematic reviews have noted the failure of interventions to achieve HIV and IPV prevention among young women in the sub‐Saharan African region, and the lack of proven successful PrEP adherence interventions [62, 63, 64, 65]. Tu'Washindi's promising results may stem from three key design strengths largely missing from previous interventions [66, 67]. First, it was designed “from the ground up,” based on formative research to understand the specific needs of AGYW in this context, and with meaningful involvement of AGYW and their service providers through a design workshop that informed selection and development of key components. The originally planned intervention was largely redesigned based on these activities [54]. Second, the intervention works at the community, partner and individual levels to simultaneously address multiple factors influencing PrEP use and vulnerability [66, 67], and to build women's agency by helping them identify a range of actions they can take to achieve their health and relationship goals, even in the context of oppressive gender norms [68]. Most existing PrEP support interventions have focused on individual‐level barriers and facilitators [63, 65]. Despite increasing calls for more holistic approaches to PrEP delivery that include community, family and partners as key influencers, few interventions working at these levels have been rigorously evaluated to date [65, 69–71]. Third, the intervention used a gender‐synchronous approach, including both separate and joint activities for men and women, incorporating findings from prior studies that working only with women can lead to male resistance and that providing opportunities for men to engage in dialogue on their own and jointly with women and girls can support behaviour change [72].

The strengths described above may also help explain how the intervention could have reduced IPV. Our primary goal in measuring IPV was to ensure that the intervention did not increase risk by encouraging AGYW to disclose PrEP use, gain partner support and use PrEP even without partner support. The trend towards reduced IPV reported in the intervention arm was an unexpected finding. In our qualitative analyses reported elsewhere, participants attributed reductions in violence to the communication skills they learned from Tu'Washindi, which helped them prevent and de‐escalate conflict, and to the PrEP education that men received through the study, which reduced their male partners’ misperceptions about PrEP and facilitated partner support for PrEP use upon disclosure [73]. Other studies have found that partner's opposition to HIV prevention method use can result in IPV, and that access to credible sources of information about HIV prevention and contraception assists in their acceptance of the product or service [22, 44, 45, 74, 75].

The Tu'Washindi intervention primarily sought to address *partner‐related* barriers to PrEP use, but support club sessions also aimed to increase knowledge, skills, self‐efficacy and social support around PrEP use for AGYW participants [54]. Despite the preliminary evidence of intervention effectiveness and its integration into adolescent‐friendly PrEP delivery at the DREAMS Safe Spaces, AGYW in the intervention arm had suboptimal adherence levels, suggesting the need to better address barriers, such as stigma, risk perception, side effects and pill fatigue [69, 70]. Promising approaches could include community‐wide education to reduce stigma, decision support tools and the provision of new PrEP delivery modalities, such as the ring or injectable PrEP, which may reduce user burden. Future efforts could include adapting Tu'Washindi or delivering it in conjunction with other interventions to incorporate these strategies.

This study has several limitations. First, because it was designed as a pilot, it had a small sample size and short follow‐up duration, limiting our ability to draw strong conclusions about effectiveness. Second, electronic adherence monitoring may not have accurately reflected participant's PrEP use, if they chose to store their pills in a separate container, removed several pills at once to facilitate covert use or opened the pillbox without taking the pills. Third, we did not enrol any AGYW ages 15–16 and thus cannot draw conclusions about intervention effectiveness in this age range. This was because the PrEP rollout in DREAMS focused on AGYW ages 18 and above, so few younger adolescents were using PrEP or considered themselves eligible. Finally, the intervention was developed to be delivered within DREAMS and to build upon, rather than replicate, other DREAMS interventions targeting barriers to PrEP use, such as community mobilization and norms change, youth‐friendly services and school‐based IPV and HIV education [52]. This choice was intentional, reflecting a recognition that in many real‐world settings, multiple HIV prevention programmes will be active concurrently and should ideally complement each other. However, it limits our ability to generalize results to AGYW outside of DREAMS. Many of these limitations are addressed in a larger effectiveness trial currently underway among AGYW in Siaya County [76]. That trial is powered to assess both PrEP and IPV outcomes (results are expected in 2026).

## 5 CONCLUSIONS

The preliminary evidence that Tu'Washindi increased PrEP uptake and adherence underscores the importance of designing interventions in collaboration with local stakeholders; engaging AGYW and their male partners individually and as couples; and working across socio‐ecological levels to address partner and relationship factors contributing to HIV and IPV. Given the important role of male partners in supporting or obstructing PrEP use, and the consequences of PrEP use for relationship health, future research should develop and test additional approaches to build male partner support and ensure that these approaches can be feasibly scaled up and delivered alongside ongoing programmatic efforts addressing other barriers to PrEP use and HIV prevention.

## COMPETING INTERESTS

The authors declare that they have no competing interests.

## AUTHORS’ CONTRIBUTIONS

STR conceptualized the manuscript. ENB and STR analysed the data. MH, AMM, SOO, ETM and KA contributed to the interpretation of results. SOO and KA were involved with study implementation and data collection at the site. STR wrote the original manuscript draft. All authors were involved in the study design and overall study implementation, participated in revising the manuscript and approved the final version.

## FUNDING

Research reported in this publication was supported by the National Institute of Mental Health of the National Institutes of Health under Award Number R34MH114519.

## DISCLAIMER

The content is solely the responsibility of the authors and does not necessarily represent the official views of the National Institutes of Health.

## Supporting information

Supporting Information
**Table S1**: Intervention quality and fidelity (cells contain frequencies, single values or mean and range of values)
**Table S2**: PrEP adherence outcome, stratified by PrEP use at enrolment

## Data Availability

The data that support the findings of this study are available from the corresponding author upon reasonable request.
