# Supplementary material for: Breaking down relationship barriers to increase PrEP uptake and adherence among adolescent girls and young women in Kenya: safety and preliminary effectiveness results from a pilot cluster‐randomized trial
Source: J Int AIDS Soc. 2023 Dec 20;26(12):e26198. doi: 10.1002/jia2.26198 (PMC10733161; doi:10.1002/jia2.26198)
Supplement: Supplementary file 1 — Supporting Information Table S1: Intervention quality and fidelity (cells contain frequencies, single values or mean and range of values) Table S2: PrEP adherence outcome, stratified by PrEP use at enrolment [file JIA2-26-e26198-s001.docx]

Supplemental Table 1: Intervention Quality and Fidelity (cells contain frequencies, single values, or mean and range of values)

|  | | | | |
| --- | --- | --- | --- | --- |
|  | **Site 1** | **Site 2** | **Site 3** | **Total** |
|  | **N** | **N** | **N** | **N** |
| **1. Support Clubs** |  |  |  |  |
| Number of sessions held | 8 | 8 | 8 | 24 |
| Number expected | 8 | 8 | 8 | 24 |
| Session duration, minutes | 110 (55-141) | 116 (73-130) | 109 (70-150) | 112 (55-150) |
| Proportion of expected activities completed | 0.98 (0.89-1.0) | 0.99 (0.96-1.0) | 0.92 (0.81-1.0) | 0.97 (0.81-1.0) |
| *Quality Scores (1= poor, 5 = excellent)* |  |  |  |  |
| Session | 4.9 (4.8-5.0) | 4.7 (3.8-5.0) | 4.9 (4.6-5.0) | 4.8 (3.8-5.0) |
| Facilitators | 5.0 (4.8-5.0) | 4.9 (4.6-5.0) | 4.9 (4.2-5.0) | 4.9 (4.2-5.0) |
| Guest Speaker* | 5.0 (4.80-5.0) | 4.9 (4.6-5.0) | 5.0 (5.0-5.0) | 5.0 (4.6-5.0) |
| Overall | 5.0 (5.0-5.0) | 4.8 (4.0-5.0) | 4.5 (4.0-5.0) | 4.8 (4.0-5.0) |
| **2. Buddy Days** |  |  |  |  |
| Number of sessions held | 1 | 1 | 1 | 3 |
| Number expected | 1 | 1 | 1 | 3 |
| Total attendees | 130 | 76 | 78 | 284 |
| Couples attended | 38 | 32 | 13 | 83 |
| Session duration, minutes | 180 | 138 | 170 | 166.7 (138-180) |
| Proportion of expected activities completed | 1 | 0.76 | 1 | 0.92 (0.76-1.0) |
| *Quality Scores (1= poor, 5 = excellent)* |  |  |  |  |
| Session | 5.0 | 5.0 | 5.0 | 5.0 (5.0-5.0) |
| Facilitators | 4.8 | 5.0 | 5.0 | 4.9 (4.8-5.0) |
| Overall | 5.0 | 5.0 | 5.0 | 5.0 (5.0-5.0) |
| **3. Male Sensitization** |  |  |  |  |
| Number of sessions held | 4 | 4 | 6 | 14 |
| Number of sessions expected | 10 | 10 | 10 | 30 |
| Number of men reached total | 39 | 64 | 116 | 219 |
| Session duration, minutes | 52 (30-71) | 23 (15-35) | 53 (40-62) | 44 (15-71) |
| Proportion of expected activities completed^ | 1.0 (1.0-1.0) | 0.88 (0.75-1.0) | 1.0 (1.0-1.0) | 0.96 (0.75-1.0) |
| *Quality Scores (1= poor, 5 = excellent)^* |  |  |  |  |
| Session | 4.9 (4.75-5.0) | 4.3 (4.0-4.5) | 4.9 (4.8-5.0) | 4.7 (4.0-5.0) |
| Facilitators | 4.9 (4.8-5.0) | 4.7 (4.4-4.8) | 4.9 (4.8-5.0) | 4.9 (4.4-5.0) |
| Overall | 5.0 (5.0-5.0) | 4.5 (3.0-5.0) | 4.3 (3.0-5.0) | 4.6 (3.0-5.0) |
| **Guest speakers were present at sessions 1-5, and at session 6 at Site 1.*  ^ Proportions and scores reflect fidelity and quality at the 14 male sensitization sessions that were held. | | | | |

Supplemental Table 2: PrEP Adherence Outcome, stratified by PrEP use at enrollment

| **PrEP adherence, continuous^*§^** | | | **N** | **Mean (SD) days of use** | **Mean (SD) days opened** | **aIRR^†^** | **95% CI** | **p** | |
| --- | --- | --- | --- | --- | --- | --- | --- | --- | --- |
| **On PrEP at enrollment** | |  |  |  |  |  |  |  | |
| Number of days with Wisepill opening | | Intervention | 15 | 169.7 (48.6) | 56.4 (62.9) | 1.93 | (1.00, 3.73) | 0.05 | |
|  |  | Control | 18 | 185.9 (28.2) | 27.7 (30.0) |  |  |  | |
| **Started PrEP during the study** | |  |  |  |  |  |  |  | |
| Number of days with Wisepill opening | | Intervention | 14 | 142.2 (44.8) | 27.4 (36.2) | 2.20 | (0.94, 5.17) | 0.07 | |
|  |  | Control | 6 | 111.5 (85.9) | 8.0 (6.6) |  |  |  | |
| *Among those with any PrEP use during the study, i.e. on PrEP at enrolment or initiated during study (n=58).  **^§^** Wisepill device data are missing or uninterpretable for 5 participants; n=53 (Intervention arm n=29; control arm n=24) | | | | | | | |  |  |
| **^†^**All effect estimates are adjusted for matched pairs of study sites. The estimate for PrEP continuation is additionally adjusted for time since PrEP initiation. The estimate for the number of days of Wisepill opening is additionally adjusted for the number of days the participant was on PrEP and had a Wisepill device in her possession. | | | | | | | |  |  |
